# Differences in Al sensitivity affect establishment of Populus genotypes on acidic forest land

**DOI:** 10.1371/journal.pone.0204461

**Published:** 2018-09-26

**Authors:** Henrik Böhlenius, Håkan Asp, Karin Hjelm

**Affiliations:** 1 Swedish University of Agricultural Sciences, Department of Southern Swedish Forest Research Centre, Alnarp, Sweden; 2 Swedish University of Agricultural Sciences, Department of Biosystems and Technology, Alnarp, Sweden; 3 The Forestry Research Institute of Sweden (Skogforsk), Ekebo 2250, Svalöv, Sweden; University of Alberta, CANADA

## Abstract

Forest lands hold great potential for *Populus* plantations, but in native boreal forests, soils normally have low pH and thus higher levels of aluminum ions (Al^3+^ and hydroxides). Aluminum (Al) is one of the major factors limiting plant growth on these soils by inhibiting root growth, thus reducing water and nutrient uptake and slowing growth. There is a large variation in Al resistance both among and within species. In this study, growth responses of greenhouse-grown hybrid aspen (*P*. *tremula* × *tremuloides*) and poplar (*P*. *trichocarpa* hybrids) were monitored in relation to changes in Al concentrations. In quartz sand, hybrid aspen was more tolerant to exogenous application of Al than *P*. *trichocarpa* hybrids. This difference in Al-tolerance was further confirmed by hematoxylin staining of the roots, with hybrid aspen displaying less staining after Al treatment than poplar clones. When planted on forest land with low pH, hybrid aspen increased growth after planting and showed low mortality. This was not the case for poplar clones; plant height decreased after planting and mortality increased. Together, our results suggest that differences in initial growth and survival on forest land among hybrid aspen and the tested poplar clones may be connected to differences in Al tolerance. Our findings that staining with hematoxylin can identify Al-tolerant *Populus* genotypes may help identify Al-tolerant genotypes suitable for forest land.

## Introduction

Soil acidity limits plant growth in both forestry and agriculture. A large proportion of arable land in the world is naturally acidic, as is the boreal and nemo-boreal forest, stretching across Scandinavia, northern Russia, China, Canada and northern USA [1]. Plantations of *Populus* genotypes (hybrid aspen and hybrid poplars) are mainly located on marginal abandoned agricultural land [2–9], but can also be found on floodplains [10] and forest land [7,11,12]. Considerably more forest land is available than agricultural land. If it can be used to grow *Populus*, biomass and food production would not compete. Plantations of hybrid aspen are susceptible to extensive grazing from moose and deer, and thus require expensive fencing [13,14].

Plantations of hybrid poplar on forest land therefore offer an attractive option, but we have limited knowledge of how different genotypes respond to different soil characteristics. Boreal forest soils are acidic with pH between 3.7 and 6.4 [1].

*P*.*trichocarpa* (and their hybrids) are nutrient demanding and suffer from growth reductions when the pH are <5 [15, 16, 17, 18]. In contrast, aspen (*Populus tremula* L.) are frequently found in boreal forests where the soil pH is low [19] and quaking aspen (*Populus tremuloides L*.) have been suggested to have acidic soil resistance [20]. As soil acidity increases, the solubilization and bioavailability of aluminum (Al) increases [21]. This suggests that in order to grow on forest land, plants needs to have a certain level of Al tolerance and that the species might have different level of Al tolerance and thus different ability to grow in acidic soils.

The sensitivity of poplars to Al may disqualify most of the available boreal and nemo-boreal forest land for poplar production. Understanding the mechanisms of Al tolerance in hybrid aspen and poplars is critical for plant establishment and future stand development on these sites. Thus, there is a need for complimentary studies of differences in Al tolerance thresholds in hybrid aspen and hybrid poplars and how this influences growth on forest land during the first years of establishment.

At high concentrations Al is toxic, inhibiting root growth [22–24]. Thus, Al is one of the major chemical constraints on crop yield and forest productivity [25, 26] on acidic soils. Al toxicity is a result of cationic Al binding to the cell wall, membranes and metabolites [25, 27]. Cell wall binding of Al causes poor root growth [28,29], with the root apex being the most sensitive region to Al-induced stress [30, 31]. Al-membrane interactions also change nutrient-uptake processes [32,33] and alters ion fluxes and membrane channel activity [33,34].

The mechanism of Al sensitivity and resistance has been well documented for agricultural crops and tree species [35,36] and for genotypes of poplar species [37,38]. These plants show great diversity in their response to Al. *Populus* exudes organic acids that inhibit Al uptake by roots. Indicator dyes such as hematoxylin have proven useful in identifying Al-tolerant genotypes in a many species (barley, wheat, tomato, teak and poplar) [39–43]. However, whether these dyes can detect Al-sensitive *Populus* genotypes is unclear. Thus, development of non-destructive bio-marker methods like hematoxylin staining to detect Al-sensitivity is needed.

The aim of this study was to investigate differences in Al-sensitivity among hybrid aspen and hybrid poplars (*P*. *trichocarpa* hybrids) and how this affects their growth on forest soils. First, we assessed Al tolerance of hybrid aspen and hybrid poplars in a greenhouse study complemented by root staining with hematoxylin as an indicator of Al-sensitivity. Secondly, we explored how the same hybrid aspen and hybrid poplar clones could establish at two contrasting forest sites. Our overarching hypothesis was that differences in Al-sensitivity among hybrid aspen and hybrid poplars impact growth potential on forest sites.

## Materials and methods

### Plant material

We selected three poplar clones (OP42: *P*. *trichocarpa × P*. *maximowiczii*, Rochester: *P*. *nigra × P*. *maximowiczii*, and clone 14: *P*. *trichocarpa* (here after named poplar) and one hybrid aspen clone (S21K884012: *P*. *tremula* ×*P*. *tremuloides*) for this study. These were chosen for their commercial availability, good rooting capacity, growth performance and genetic diversity. The hybrid aspen clone originates form a breeding program performed by the Swedish match company in the 1940s. From theses crosses, clones were selected and tested by the Forestry Research Institute of Sweden at the end of 1980 and the used clone are one of the best performing hybrid aspen clones. The poplar clone OP42 and Rochetser were bred in 1924 by the Oxford Paper Company, USA and Clone 14 is of unknown origin but belongs to the commercial clones from the Forestry Research Institute of Sweden.

### Experimental design, growth conditions and data collection for analysis of Al-sensitivity

To produce rooted poplar plants, dormant cuttings were collected in January and stored at 4°C until the start of the experiment. The cuttings had three buds, and were about 10 cm long by 10 mm diameter. Before the Al treatments, root-washed container-grown hybrid aspen (30–40 cm tall, 3.5–4.0 mm root collar diameter, purchased at Svenska skogsplantor, Hallsberg, Sweden) and poplar cuttings were planted in 3-liter pots containing siliceous quartz sand (0.45 mm grain size) purchased from Baskarpssand AB, Habo, Sweden. Poplar and hybrid aspen plants were kept in the greenhouse (temperature set to 20°C and 20 h of additional light supplied from fluorescent lamps with total photon flux of 130 μmol m^−2^ s^−1^) for three weeks, where they initiated root development and grew about 20 cm taller. During this time, plants were irrigated with nutrient solution prepared by dissolving solid fertilizer (0.37 g Superba röd and 0.37 g calcinit YARA Liva per liter deionized water) and adjusting the pH to 4.2 with hydrochloric acid (HCl). At the start of the experiment, plants were placed in 7 blocks where each block contained one plant per genotype and Al treatment. The Al treatment were performed by irrigating with nutrient solution supplemented with AlCl_3_ to achieve (Al) 0, 10, 30, 50, 100, 200 and 300 mg /l and pH was adjusted with sodium hydroxide (NaOH) to 4.2. After six weeks of Al treatment, plant heights were measured and stems, leaves and roots were weighed after drying at 70°C for 48 hours.

### Hematoxylin staining

The same hybrid aspen and poplar clones described above were stained with hematoxylin after treatment with Al in nutrient solution. First, poplar cuttings and root-washed hybrid aspen plants were pre-grown in the nutrient solution described above for two weeks to initiate root and shoot growth. After this pre-growth, plants were transferred to a nutrient solution containing either 0, 10 or 20 mg/l Al for 24 h. To prepare the treatment solutions, nutrient solution was supplemented with AlCl_3_ on the day of use. All solutions were adjusted to pH 4.2 with NaOH (10 and 20 mg/l Al) or HCl (0 mg/l Al). After Al treatment, roots were washed with deionized water for 15 minutes and stained with 1g/l hematoxylin (Sigma–Aldrich, Seelze, Germany) and 0.1g/l of KIO_3_ (Riedel-de Haën, St: Louis USA) for 15 min. After staining, roots were washed again for 20 minutes to remove excess stain according to [44]. The staining was repeated twice with similar results. The degree of staining was determined by ocular inspection.

### Forest experiment—Site description and soil treatment

The field experiment used two forest sites in the southernmost part of Sweden: Tönnersjö (56°42'7.0"N 13°6'21.4"E) and Sävsjöström (56°59'5.6"N 15°28'55.3"E). Both sites were clear-felled in winter 2012. The forests differ in site index; at Tönnersjö, spruce reaches a dominant height of 34 m after 100 years (G34) with a growth of 12.6 m^3^ ha^-1^ year^-1^ and at Sävsjöström, pine achieves a dominant height of 22 m at 100 years (T22) with a growth of 5.1 m^3^ ha^-1^ year^-1^). Mean annual precipitation and temperature are about 1000 mm and 8°C at Tönnersjö and 800 mm and 5°C at Sävsjöström [45]. At these sites, the mineral soil pH was approximately 4.8 at Tönnersjö and 4.7 at Sävsjöström while the extractable aluminum concentration in the soil was approximately 260–330 mg/kg soil (dry matter) according to the Swedish national survey of forest soils and vegetation [46]. Tönnersjö was dominated by spruce (*Picea abies* (L). Karst) and Sävsjöström was dominated by Scots pine (*Pinus sylvestris* L.) prior to cutting. The ground vegetation on the clearcuts was dominated by grass (*Deschampsia*.*sp*). To reduce browsing by deer and moose, the experimental areas were fenced. Both sites have podzolic moraine soils. Inverse soil scarification was conducted with an excavator the same spring as planting was performed. Bare-rooted poplar plants and container-grown hybrid aspen plants were purchased from Svenska Skogsplantor, Hallsberg, Sweden. Planting was undertaken in May 2012. Each site was divided into four blocks (8 m x 4 m). In each block, eight plants of each genotype were manually planted 1 m apart in the scarified rows. Total plant height and plant survival were recorded at planting and in October following the first and second growth periods. The land owner has gave their permission to conduct the field experiments at both sites Tönnersjö and Sävsjöström. No other specific permissions were needed in order to conduct the experiments as the experiment did not involve protected or endangered species.

### Statistical analyses

All analyses described here were implemented in R version 3.1.1 [47]. To test the effects of Al treatments on growth and survival or height at the field experiment, we used mixed models implemented in the ‘lme4’ package. Relative height growth and biomass were calculated by dividing the height or biomass for individual plants at each Al treatment with the mean value of untreated plants of the same genotype. For the Al-treatment study the response variables tested were relative height and relative biomasses of leaves, stems and roots. Al-treatment and genotype (hybrid aspen and poplar clones) were set as fixed effects while block was treated as a random effect. All poplar and hybrid aspen clones were compared to each other at the different Al concentrations. For the field experiment, total plant height and survival were the response variables. Genotype (hybrid aspen and poplar clones) was treated as a fixed effect and block as a random effect.

The models used for the greenhouse experiments was;

$$y_{ij}=Clone_{j}+b_{i}+e_{ij}$$

where *y*_*ij*_ is the observed heights and survival, *clone*_*j*_ is the fixed effect of the clones used, *b*_*i*_ is the random effect of block, and *e*_*ij*_ is the random error term assumed to be normally distributed with mean 0 and constant variance. The subscripts represents *j* = 1-4for clone and *i = 1–7* for block.

The models used for the field experiments was;

$$y_{ijk}=Clone_{ik}+b_{i}+e_{ijk}$$

where *y*_*ijk*_ is the observed heights and survival, *clone*_*i*_ is the fixed effect of the clones used, *b*_*i*_ is the random effect of block, and *e*_*ijk*_ is the random error term assumed to be normally distributed with mean 0 and constant variance. The subscripts represents *j = 1–4* for clone, *i = 1–4* for block, *k* replication within block and clone.

The two sites, Sävsjöström and Tönnersjö differ in geographical location with different temperature, precipitation and site index and were therefore analyzed separately. Survival and height of all poplar and hybrid aspen clones was compared to each other. To evaluate differences among treatments, we used Tukey´s HSD as a post-hoc test, implemented in the “lsmeans” R package. A *p*-value of 0.05 was used as the cutoff for statistical significance. Residuals were inspected and showed normal distributions with no high-leverage outliers.

## Results

### Analysis of Al tolerance among hybrid aspen and poplars under controlled greenhouse conditions

**Fig 1:**
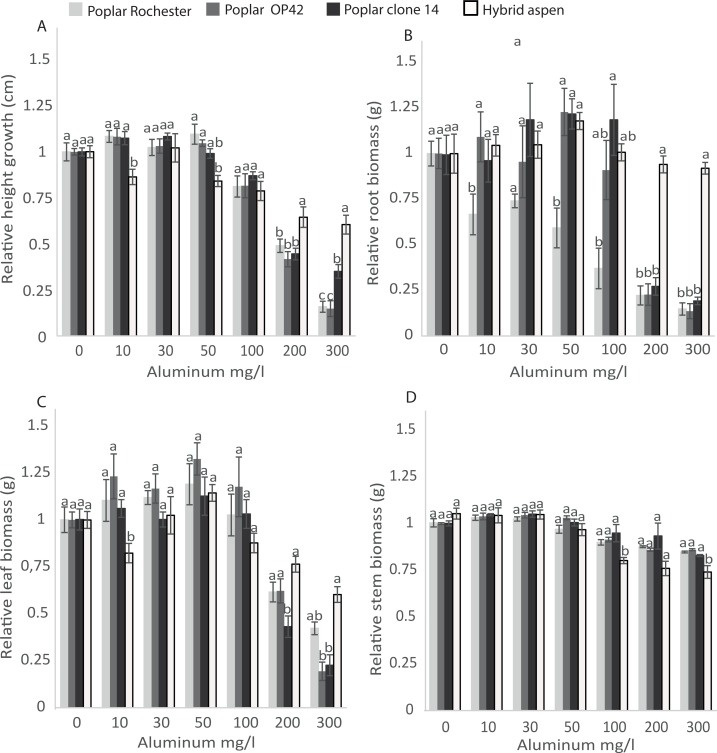
Impact of different Al concentrations on hybrid aspen and three poplar clones. Relative height growth (A), relative biomasses of root (B), leaf (C) and stem (D) were determined six weeks after planting of hybrid aspen and poplar; OP42 *P*. *trichocarpa × P*. *maximowiczii*, Rochester *P*. *nigra × P*. *maximowiczii* and clone 14 *P*. *trichocarpa*. Data shown are mean values of seven blocks (*n* = 7). Error bars show standard errors. Bars labeled with different letters are significantly different at the *p =* 0.05 level within each Al concentration treatment.

When grown in forest soils of different pH, hybrid aspen is less sensitive to changes in soil pH wile *P*.*trichocarpa* prefers pH > 5 and suffers from growth reduction when pH < 5 [15, 16, 17, 18]. One of the mineral ions that increases with low soil pH and can have negative effect on plant growth is Al^3+^. To investigate if this difference in tolerance against low pH are connected to different sensitivity against Al, we treated poplar and hybrid aspen plants with different Al concentrations but at the same pH in an inert growth medium (sand) under controlled greenhouse conditions. The hybrid aspen and poplar clones differed greatly in Al sensitivity. The relative root biomass and relative height growth were lower for all poplar clones at 200 and 300 mg Al/l (Fig 1A and 1B). We also found that relative root biomass for Rochester (*P*. *nigra* × *P*. *maximowiczii*) was lower than hybrid aspen at Al concentrations of 10 and 50 mg/l. For relative leaf and stem biomasses, differences among poplar clones and hybrid aspen were not as clear but there was a tendency towards lower relative leaf biomass for clone 14 at 200 and 300 mg Al/l and for OP42 at 300 mg Al/l (Fig 1C). For stem biomass, no significant differences among poplar clones and hybrid aspen were found for 10, 30, 50, or 200 mg/l (Fig 1D). In summary, this demonstrates that there are differences in Al sensitivity among hybrid aspen and the tested poplar clones, but among the poplar clones no differences were found.

### Roots hematoxylin staining after treatment with Aluminum

When plants are exposed to Al, sensitive genotypes exhibit inhibition of root growth [22–24] with root apex being the most sensitive region to Al induced stress [33, 34]. Staining with different dyes have been used in cereal crops, to identify tolerant genotypes [39–43] were less staining is related to higher Al tolerance. To further collaborate the observed Al sensitivity among the tested *Populus* genotypes and develop a non-destructive method for detecting tolerant and sensitive *Populus* genotypes, plants were grown in nutrient solution, treated with Al and stained with hematoxylin.

**Fig 2:**
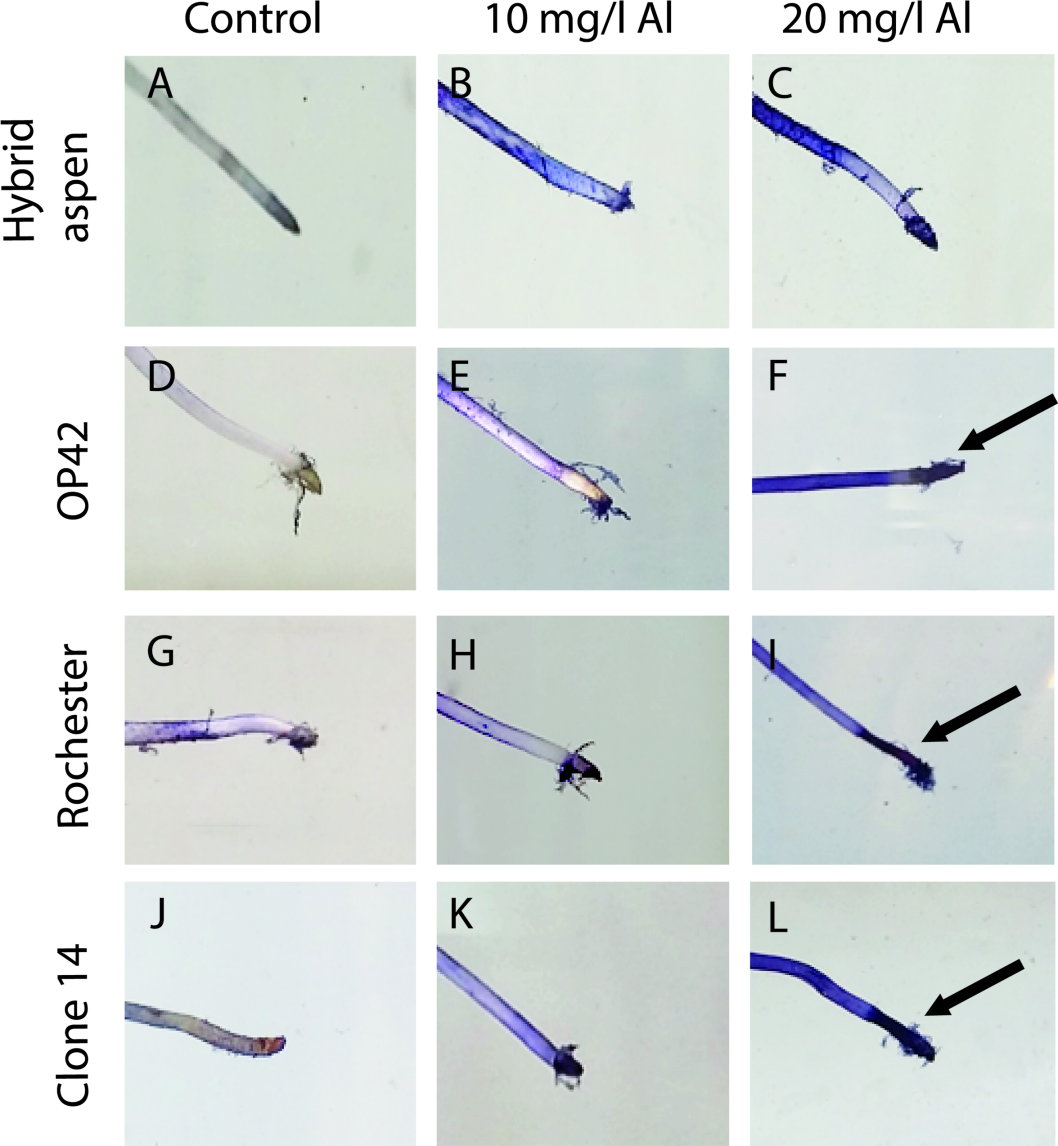
Hematoxylin staining of poplar and hybrid aspen roots. Plants were grown in nutrient solutions and treated with Aluminum (Al) concentrations of 0, 10 and 20 mg/l. Analyzed genotypes were hybrid aspen (A) and hybrids OP42 *P*.*trichocarpa x P*. *maximowiczii* (B), Rochester *P*. *nigra × P*. *maximowiczii* (C) and clone 14 *P*.*trichocarpa* (D). Arrows highlight strong hematoxylin staining (blue) at the root apex.

The Al sensitivity of hybrid aspen and poplar clones was reflected in differential Al-induced hematoxylin staining (Fig 2A–2L). Hematoxylin staining increased with Al concentration in the nutrient solution but differed among the genotypes. Hybrid aspen produced less hematoxylin staining than the poplar clones (Fig 2C, 2F, 2I and 2L) but among the poplar clones no obvious difference in hematoxylin staining were noted. Repetition of the staining test showed similar results.

### Survival and early growth on forest land

**Fig 3:**
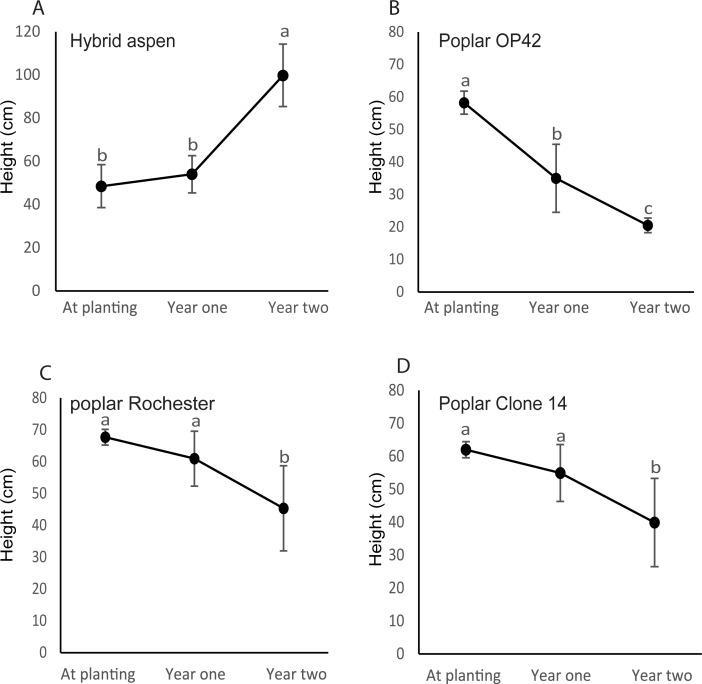
**Height of hybrid aspen (A) and poplars (B-D) at forest site Tönnersjö.** Plant heights were recorded at planting and after the first and second growth periods. Mean values with the same letters are not significantly different at *p* = 0.05. Error bars indicate standard errors (n = 4).

**Fig 4:**
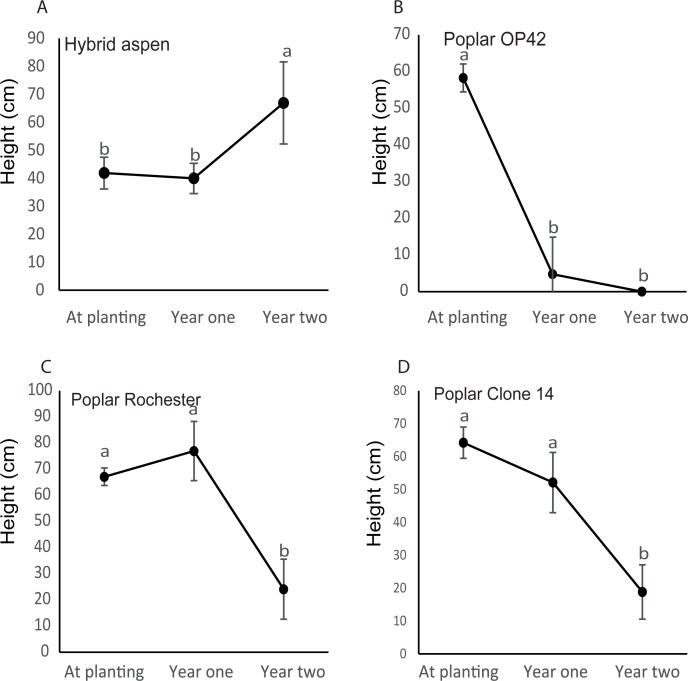
**Height of hybrid aspen (A) and poplars (B-D) at forest site Sävsjöström.** Plant heights were recorded at planting and after the first and second growth periods. Values with the same letters are not significantly different at *p* = 0.05. Error bars in A, C and D indicate standard errors (n = 4). In B, error bars indicate standard errors (n = 4).

To give further insight to if Al- sensitivity could influence survival and early growth, hybrid aspen and the poplar clones were planted in field experiments at two different forest sites. The forest soils are typically characterized by low pH and high Al concentrations [1, 46]. At experimental site Tönnersjö, hybrid aspen and poplar clones had similar survival after the first growing period (Table 1), but at Sävsjöström, *P*. *trichocarpa* hybrids had lower survival than hybrid aspen (Table 2). After the second year, survival of poplar clones was lower than for hybrid aspen at both sites. Hybrid aspen plants grew taller two years after planting at both experimental sites (Fig 3A and Fig 4A). This was not the case for poplar. Instead, height for all the clones were decreased (Fig 3B to 3D and Fig 4B to 4D). The reduction in height of the poplar clones was caused by a die back of the leading shoot resulting in a new shoot developing from lower parts of the stem. In most cases, this shoot was in turn damaged, which lead to development of a new shoots at even lower parts of the stem. (Fig 3B to 3D and Fig 4B to 4D).

**Table 1:** Analysis of plant survival (percent) at experimental site Tönnersjö after the first and second year after planting.

Genotype		Year one				Year two			
*Hybrid aspen*		88	±	18	a	88	±	18	a
S21K88012									
*Poplar*									
Clone 14		91	±	12	a	34	±	16	b
Rochester		88	±	13	a	50	±	13	b
OP42		81	±	18	a	50	±	18	b

Note; the data shown are mean values standard deviation (n = 4). Analyzed genotypes are hybrid aspen and poplars, OP42: *P*.*trichocarpa x P*.*maximowiczii*, Rochester: *P*.*nigra x P*.*maximowiczii* and clone 14: *P*.*trichocarpa*.

**Table 2:** Analysis of plant survival (percent) at experimental site Sävsjöström after the first and second year after planting.

		Year one				Year two			
*Hybrid aspen*		94	±	13	a	88	±	10	a
S21K88012									
*Poplar*									
Clone 14		36	±	20	b	27	±	17	b
Rochester		36	±	13	b	27	±	18	b
OP42		13	±	14	b	9	±	18	b

Note; the data shown are mean values standard deviation (n = 4). Analyzed genotypes are hybrid aspen and poplars, OP42: *P*. *trichocarpa x P*. *maximowiczii*, Rochester: *P*. *nigra x P*. *maximowiczii* and clone 14: *P*. *trichocarpa*.

## Discussion

Reduction of growth is often observed when Al-sensitive species and genotypes are exposed to Al. Often, reduced root growth is a good indicator of Al sensitivity, reflecting Al-induced root damage, which in turn hampers plant growth [25–27]. In the current study, four *Populus* genotypes displayed distinct differences in Al-sensitivity based on relative root biomass and height growth (Fig 1A and 1B). This was not as pronounced for leaf biomass or stem biomass (Fig 1C and 1D). Our results are in line with other studies of *Populus*, which suggest that both root and shoot growth are useful to investigate Al sensitivity [37]. We found that the hybrid aspen was more tolerant than the tested poplars with little difference among the tested poplar clones. Although the number of *Populus* genotypes used in this study was relatively limited, a clear difference in Al-sensitivity could be detected among hybrid aspen and all poplar clones.

The experimental design used in this study does not allow us to determine if increased Al tolerance in hybrid aspen comes from *P*. *tremula* or *P*. *tremuloides*. Soils, where *P*. *tremula* grows, are usually acidic with values ranging from 3.7 to 6.4 [1] [19], and *P*. *tremuloides* has also been suggested to have acidic soil resistance [20]. *P*. *tremula* is native to Sweden, and has coevolved with the soil conditions at forest sites possibly facilitating high Al tolerance but *P*. *tremuloides* has also been suggested to have acidic soil resistance [20] that could result in higher Al tolereance. Despite our failure of determine the source of Al tolerance, i.e. *P*.*tremula* or *P*.*tremuloide*, our results give insight at what level of Al tolerance that might be needed in order for a poplar clone to grow at acidic forest sites in Sweden. *Populus trichocarpa* (and their hybrids) are usually nutrient demanding [15], and suffer from growth reduction when the pH is <5 [15, 16, 17, 18] but there are large differences in Al tolerances between clones and poplar genotypes [37,38].

Our finding that there is differences in Al sensitivity among *Populus* genotypes agrees with other studies showing a large variability in Al sensitivity among poplar genotypes [37,38]. The higher Al concentrations (>100 mg/l) resulted in distinct root and height growth differences in the tested hybrid aspen and poplar clones (Fig 1A and 1B). However, we could detect significant differences in relative root growth at 10 mg/l for the Rochester poplar clone, but not for height growth or stem and leaf biomass. This suggests that more than one growth parameter should be used to determine Al-sensitivity. Our results also suggest that a dose response setup is needed for initial studies of Al sensitivity to capture the Al-sensitivity threshold similar to those suggested by Barceló J and Poschenrieder C (2002) [48]. Similar to Smith E, et al (2011) [37], our results suggests that shoot growth can be used to capture Al-sensitivity. However, our data suggest that both height growth and root biomass reflect Al-sensitivity for poplar genotypes and thus could be used as indicators. The observed change in height growth could be a result of reduced nutrient uptake [20,49] and that in Al-sensitive genotypes, Al accumulated in the roots is restraining growth [49].

When planted on forest land these hybrid aspen and poplar clones showed clear differences in growth and survival. Hybrid aspen increased growth after planting (Fig 3A and Fig 4A) and survival was stable over two years at site Tönnersjö (88% year one and two) and showed minor changes at site Sävsjöström (94% to 88% for year one and two, respectively). Poplar performed differently. Instead of growing after planting, all tested clones died back at both sites. We found that for the living poplar plants, the main top shoot was often severely damaged or dead, probably due to drought and that a new dominant shoot started to grow from the lower parts of the stem but the height was shorter than for the previous leader. As a consequence, living plant heights were reduced. This clearly demonstrates that poplars can be difficult to establish on forest sites, while it is possible for hybrid aspen.

The newly-planted seedlings have limited root-system permeability, root system size, root distribution and/or root-soil contact. They are also affected by low root hydraulic conductivity, which all together can limit water uptake from the soil [50–53]. New root growth following establishment is extremely important for seedling growth [54,55]. As Al toxicity inhibits root cell division and elongation, thus reducing water and nutrient uptake this can result in poorer growth and yield [56–60]. Al toxicity also limits both rooting depth and root branching [61, 62] Al toxicity decreases drought tolerance and the use of subsoil nutrients [63]. All these processes are important for plants to initiate growth on forest land. As Al has these negative effects on root growth, Al-sensitive plants might have poor root elongation thereby having problems connecting to the surrounding soil and as a consequence, drought damage and nutrient deficiency could occur. In contrast, Al-tolerant plants have probably normal root growth and thus can connect to the surrounding soil and initiate growth.

Our results indicate that hybrid aspen with the highest tolerance to Al (Fig 1) could establish on forest land much better than the tested poplar clones (Al sensitive) that loose height from leader die back and exhibit high mortality (Figs 3 and 4 and Table 1). This suggests that tolerance of Al could be one of the factors important for establishment of *Populus* on forest land.

Although the number of poplar clones and genotypes used in this study is limited, the results do indicate that there are large differences in Al-sensitivity between the hybrid aspen clone and the poplar clones tested in this study. Generally, when planting poplar and hybrid aspen on forest sites in Sweden, hybrid aspen show a better performance (survival and growth) than poplars [64]. This is especially accentuated at sites with low pH were survival could be as low as 11% for poplar and 83% for hybrid aspen [64], results similar to ours (Tables 1 and 2). Moreover, survival and growth of hybrid aspen clones were stable, indicating that there are a common traits for hybrid aspen clones that are important for survival and growth on acidic forest land.

The mechanisms providing enhanced tolerance against Al can be divided into external and internal mechanisms. The most studied mechanism is the external exudation of organic acids. The anion of the organic acids effectively chelates Al, thereby detoxify it in the rhizosphere. For *Populus* genotypes, Al induces exudation of citrate, malate and oxalate in *P*. *tremuloides* and P. *trichocarpa* [65] and stimulates release of oxalate and citrate in *P*. *tremula* [66]. The genes responsible for the Al-induced efflux of malate and citrate have been isolated in a number of species including aspen (*Populus tremula*) [67, 68]. Recently, genes involved in cell wall modification, ion transport and oxidative stress were shown to be up-regulated during Al treatments of aspen. Among these genes, two genes that may play a role in Al-tolerance were identified, Al tolerance gene ALS3 and MATE which encodes a citrate efflux transporter [67].Thus, the observed differences in Al-sensitivity among hybrid aspen and *P*. *trichocarpa* clones or hybrids might be related to different ability to exudate organic acids, possibly citrate, to prevent Al uptake by the roots, thereby avoiding root damages. In line with this, hybrid aspen accumulated lower levels of Al than poplar (*P*. *trichocarpa*) when grown in acidic soils [16]. However, the Al tolerance could also be connected to differences in Al induced changes in the root cell wall or oxidative stress. It is possible that forest trees adapted to naturally acidic soil conditions have developed mechanisms that enables them to tolerate high Al conditions. For instance, birch (*Betula pendula*) showed no reduction in root growth at Al concentrations between 8 to 80 mg/l and for aspen (*Populus tremula*) Al concentrations higher than 7 mg/l reduced root growth [67,69]. Therefore, depending on the origin of the tree species, different Al tolerance might be found even if they are naturally occurring at forest sites.

If poplars are to be grown on forest land, clones or genotypes with similar Al tolerance as hybrid aspen should be identified. To do this, a non-destructive and efficient screening method is needed. We found that hematoxylin staining could be used to differentiate Al sensitive (poplar) from Al tolerant (hybrid aspen) genotypes as hematoxylin staining was more pronounced in Al-sensitive poplar clones than in the Al-tolerant hybrid aspen. Similar results have also been found in barley [37], wheat [3,38,39], sorghum [70] and teak [43]. Identification of tolerant poplar clones and/or genotypes could solve some of the problems establishing poplar on forest land. If such large screening of Populus genotypes/clones would be undertaken, our results indicate that the screening process might be divided into two steps. The first step would involve growing plants in nutrient solution, followed by Al treatment (20 mg/L) and staining with hematoxylin. At this step, many clones of different genotypes could be easily be tested and clones with low staining (tolerant) could be selected for further confirmation of Al tolerance. However, clones that have pore rooting might be discarded not because of their Al tolerance/sensitivity but because of the lack of root growth. During the second step, the selected clones could be grown in sand and irrigated with nutrient solution containing Al. Our results suggest that if a similar tolerance as the tested hybrid aspen clone are needed, approximately 100 mg/L Al in the nutrient solution could be used for this selection. Several other studies have demonstrated that there are differences in tolerance against Al, with at least in part from species in section *Tacamahaca* genotypes being more tolerant [38]. Thus, the tested clones in our study belongs to this group and this might be the reason for our failure of detecting clear differences among the poplar clones. However, even within the same species in this section there are clonal differences in Al tolerance [37, 38], so if poplar clones with similar tolerance as hybrid aspen are to be identified, it will probably come down to individual clones.

In conclusion, we have demonstrated that there are differences in Al sensitivity among hybrid aspen and poplar and that this correlates with difficulties in establishment on forest land. The use of hematoxylin staining to detect Al-tolerant genotypes could be an important tool to expand poplar plantations on forest land. However, this needs to be addressed in more detail to explore which poplar genotypes can cope with the high Al-levels found in acidic forest soils.
